# A dimeric zinc(II) complex: bis­[μ-1,2-bis­(1,2,4-triazol-4-yl)ethane-κ^2^ *N* ^1^:*N* ^1′^]bis­[dinitritozinc(II)]

**DOI:** 10.1107/S1600536810034203

**Published:** 2010-09-04

**Authors:** Rongxian Zhang, Qiuyun Chen, Jing Gao, Xiangyang Wu

**Affiliations:** a School of Chemistry and Chemical Engineering, Jiangsu University, Zhenjiang 212013, People’s Republic of China; b School of Pharmacy, Jiangsu University, Zhenjiang 212013, People’s Republic of China

## Abstract

The coordination geometry of the Zn^II^ atom in the title complex, [Zn_2_(NO_2_)_4_(C_6_H_8_N_6_)_2_], is distorted octa­hedral, in which the Zn^II^ atom is coordinated by two N atoms from the triazole rings of two symmetry-related 1,2-bis­(1,2,4-triazol-4-yl)ethane ligands and four O atoms from two nitrite ligands. Two Zn^II^ atoms are bridged by two organic ligands, forming a centrosymmetric dimer. Weak C—H⋯N and C—H⋯O hydrogen bonds play an important role in the inter­molecular packing.

## Related literature

For background to 1,2,4-triazole and its derivatives, see: Haasnoot (2000 ▶). For a related structure, see: Habit *et al.* (2009 ▶). For hydrogen bonding, see: Mascal (1998 ▶).
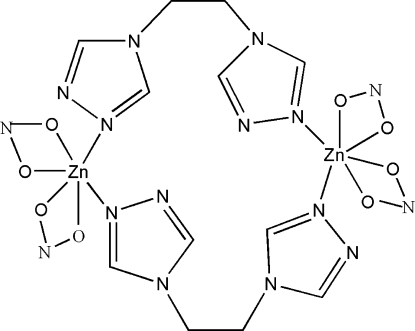


## Experimental

### 

#### Crystal data

[Zn_2_(NO_2_)_4_(C_6_H_8_N_6_)_2_]
*M* *_r_* = 643.15
Monoclinic, 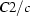

*a* = 20.491 (4) Å
*b* = 6.7087 (13) Å
*c* = 17.289 (4) Å
β = 97.125 (5)°
*V* = 2358.3 (8) Å^3^
*Z* = 4
Mo *K*α radiation
μ = 2.11 mm^−1^
*T* = 293 K
0.60 × 0.20 × 0.20 mm

#### Data collection

Rigaku Mercury CCD diffractometer
Absorption correction: multi-scan (Blessing, 1995 ▶, 1997 ▶) *T*_min_ = 0.364, *T*_max_ = 0.678
10892 measured reflections
2144 independent reflections
1945 reflections with *I* > 2σ(*I*)
*R*_int_ = 0.036

#### Refinement

*R*[*F* ^2^ > 2σ(*F* ^2^)] = 0.040
*wR*(*F* ^2^) = 0.104
*S* = 1.06
2144 reflections
172 parameters
H-atom parameters constrained
Δρ_max_ = 0.47 e Å^−3^
Δρ_min_ = −0.61 e Å^−3^

### 

Data collection: *CrystalClear* (Rigaku, 2000 ▶); cell refinement: *CrystalClear*; data reduction: *CrystalClear*; program(s) used to solve structure: *SHELXS97* (Sheldrick, 2008 ▶); program(s) used to refine structure: *SHELXL97* (Sheldrick, 2008 ▶); molecular graphics: *SHELXTL* (Sheldrick, 2008 ▶); software used to prepare material for publication: *SHELXTL*.

## Supplementary Material

Crystal structure: contains datablocks global, I. DOI: 10.1107/S1600536810034203/bv2153sup1.cif

Structure factors: contains datablocks I. DOI: 10.1107/S1600536810034203/bv2153Isup2.hkl

Additional supplementary materials:  crystallographic information; 3D view; checkCIF report

## Figures and Tables

**Table 1:** Selected bond lengths (Å)

Zn1—N4^i^	2.002 (3)
Zn1—O1	2.031 (3)
Zn1—N1	2.036 (3)
Zn1—O3	2.046 (3)
Zn1—O2	2.477 (3)
Zn1—O4	2.488 (3)

Symmetry code: (i) 
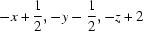
.

**Table 2:** Hydrogen-bond geometry (Å, °)

*D*—H⋯*A*	*D*—H	H⋯*A*	*D*⋯*A*	*D*—H⋯*A*
C1—H1*B*⋯O2^ii^	0.97	2.53	3.396 (5)	149
C2—H2*A*⋯O1^iii^	0.97	2.49	3.417 (4)	160
C3—H3*A*⋯O2^ii^	0.93	2.66	3.412 (5)	139
C6—H6*A*⋯N2^iv^	0.93	2.39	3.314 (4)	176

Symmetry codes: (ii) 
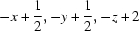
; (iii) 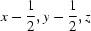; (iv) 
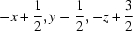
.

**Fig. 1.**
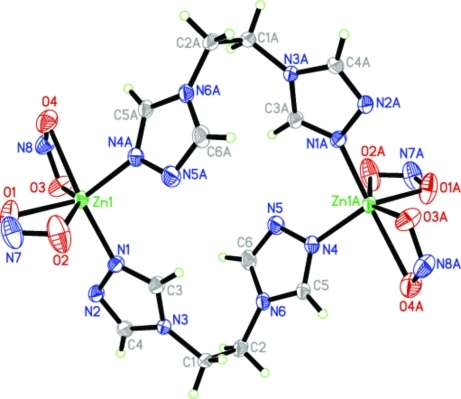
A dimeric structure of (I), with displacement ellipsoids drawn at the 30% probability level. [Symmetry code: A -x + 1/2, -y - 1/2, -z21.]

**Fig. 2.**
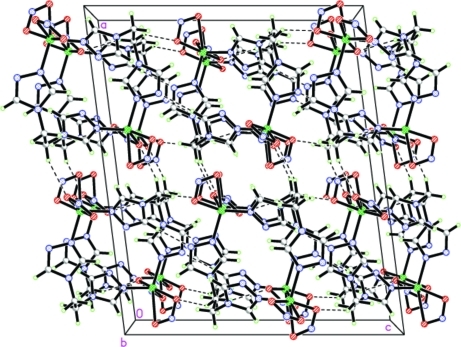
The cell packing of (I) along [010] direction.

## supplementary crystallographic information

### Comment

1,2,4-Triazole and its derivatives are very interesting ligands, because they combine the coordination geometry of both pyrazole and imidazole with regard to the arrangement of their three heteroatoms. A large number of mononuclear, oligonuclear and polynuclear transition metal complexes of 1,2,4-triazole derivatives have been synthesized and characterized because of their magnetic properties and novel topologies (Haasnoot, 2000).

In the present work, we report here the preparation and crystal structure of a dimeric zinc^II^ complex, namely, [Zn(btre)NO_2_)_2_]_2_ (I) (btre = 1,2-bis(1,2,4-triazol-4-yl)ethane.

In the complex (I), the coordination geometry of the zinc^II^ atom in the title complex, [Zn(C_6_H_8_N_6_)(NO_2_)_2_]_2_ or [Zn(btre)(NO_2_)_2_]_2_, where btre is µ-[1,2-bis(1,2,4-triazol-4-yl)ethane], is a distorted octahedron, in which the Zn^II^ atom is coordinated by two N atoms from the triazole rings of two symmetry-related btre ligands and four O atoms from two NO_2_^-^ ligands. Two Zn^II^ atoms are bridged by two organic ligands to form a dimer. Each NO_2_^-^ anion acts as a chelating coordination mode.

The crystal structure of (I) is built up from a neutral dimeric metallocycle. The dimer is centrosymmetric. As shown in Fig.1, in each dimer, two zinc^II^ centres are connected by two btre ligands resulting in a discrete Zn_2_(btre)_2_ 18-membered binuclear metallocycle.

Each zinc^II^ centre is six-coordinated by two N atoms of btre ligands and four O atoms from two NO_2_^-^ ligands (Table 1), forming a distorted octahedral geometry. Each btre exhibits a *gauche* conformation in (I). The N3—C1—C2—N6 torsion angle is 64.9 (4)°. The dihedral angle between the two triazole ring is 40.1 (2)°. The Zn···Zn separation *via* the bridging btre ligand is 7.809 (2)Å in (I), compared with the corresponding values 7.8750 (2)Å in [Zn(btre)Cl_2_]_2_ and 7.7980 (5)Å in [Zn(btre)I_2_]_2_ (Habit *et al.*, 2009).

Weak hydrogen bonds play an important role in the formation of the crystal structure. The intermolecular packing is organized by C—H···N and C—H···O hydrogen bonds (Table 2 and Figure 2; Mascal 1998).

### Experimental

A 10 ml aqueous solution of Zn(NO_2_)_2_ (1 mmol) was added to a tube, and a 10 ml MeOH solution of 1,2-bis(1,2,4-triazol-4-yl)ethane (btre) (1.0 mmol) was carefully added above the aqueous solution. Colourless crystal were obtained after about two weeks. Anal. Calcd. for C_12_H_16_N_16_O_8_Zn_2_: C, 22.41; H, 2.51; N, 34.85%. Found: C, 22.36; H, 2.44; N, 34.69%.

### Refinement

H atom were placed in idealized positions and refined as riding, with C—H distances of 0.93 (triazole) and 0.97Å (ethane), and with *U*_iso_(H) = 1.2*U*_eq_(C).

### Crystal data

**Table:**

[Zn_2_(NO_2_)_4_(C_6_H_8_N_6_)_2_]	*F*(000) = 1296
*M**_r_* = 643.15	*D*_x_ = 1.811 Mg m^−^^3^
Monoclinic, *C*2/*c*	Mo *K*α radiation, λ = 0.71070 Å
Hall symbol: -c 2yc	Cell parameters from 4261 reflections
*a* = 20.491 (4) Å	θ = 3.2–25.4°
*b* = 6.7087 (13) Å	µ = 2.11 mm^−^^1^
*c* = 17.289 (4) Å	*T* = 293 K
β = 97.125 (5)°	Block, colorless
*V* = 2358.3 (8) Å^3^	0.60 × 0.20 × 0.20 mm
*Z* = 4	

### Data collection

**Table:**

Rigaku Mercury CCD diffractometer	2144 independent reflections
Radiation source: fine-focus sealed tube	1945 reflections with *I* > 2σ(*I*)
graphite	*R*_int_ = 0.036
ω scans	θ_max_ = 25.3°, θ_min_ = 3.2°
Absorption correction: multi-scan (Blessing, 1995, 1997)	*h* = −24→24
*T*_min_ = 0.364, *T*_max_ = 0.678	*k* = −7→8
10892 measured reflections	*l* = −20→20

### Refinement

**Table:**

Refinement on *F*^2^	Primary atom site location: structure-invariant direct methods
Least-squares matrix: full	Secondary atom site location: difference Fourier map
*R*[*F*^2^ > 2σ(*F*^2^)] = 0.040	Hydrogen site location: inferred from neighbouring sites
*wR*(*F*^2^) = 0.104	H-atom parameters constrained
*S* = 1.06	*w* = 1/[σ^2^(*F*_o_^2^) + (0.0627*P*)^2^ + 2.1848*P*] where *P* = (*F*_o_^2^ + 2*F*_c_^2^)/3
2144 reflections	(Δ/σ)_max_ = 0.001
172 parameters	Δρ_max_ = 0.47 e Å^−^^3^
0 restraints	Δρ_min_ = −0.61 e Å^−^^3^

### Special details

**Table:**

Geometry. All e.s.d.'s (except the e.s.d. in the dihedral angle between two l.s. planes) are estimated using the full covariance matrix. The cell e.s.d.'s are taken into account individually in the estimation of e.s.d.'s in distances, angles and torsion angles; correlations between e.s.d.'s in cell parameters are only used when they are defined by crystal symmetry. An approximate (isotropic) treatment of cell e.s.d.'s is used for estimating e.s.d.'s involving l.s. planes.
Refinement. Refinement of *F*^2^ against ALL reflections. The weighted *R*-factor *wR* and goodness of fit *S* are based on *F*^2^, conventional *R*-factors *R* are based on *F*, with *F* set to zero for negative *F*^2^. The threshold expression of *F*^2^ > σ(*F*^2^) is used only for calculating *R*-factors(gt) *etc*. and is not relevant to the choice of reflections for refinement. *R*-factors based on *F*^2^ are statistically about twice as large as those based on *F*, and *R*- factors based on ALL data will be even larger.

### Fractional atomic coordinates and isotropic or equivalent isotropic displacement parameters (Å^2^)

**Table:**

	*x*	*y*	*z*	*U*_iso_*/*U*_eq_	
Zn1	0.377036 (17)	0.06273 (5)	0.900523 (19)	0.03325 (17)	
O1	0.42015 (16)	0.3236 (5)	0.8755 (2)	0.0767 (10)	
O2	0.3646 (2)	0.3909 (5)	0.96266 (18)	0.0876 (12)	
O3	0.40736 (13)	−0.1027 (4)	0.81217 (16)	0.0581 (7)	
O4	0.48542 (14)	−0.1050 (5)	0.90120 (16)	0.0662 (8)	
N1	0.28080 (13)	0.0744 (4)	0.85410 (15)	0.0353 (6)	
N2	0.26438 (14)	0.0921 (4)	0.77408 (15)	0.0401 (7)	
N3	0.17452 (13)	0.0807 (4)	0.83012 (15)	0.0325 (6)	
N4	0.12627 (14)	−0.4368 (4)	0.99508 (15)	0.0353 (6)	
N5	0.17231 (16)	−0.4949 (5)	0.94714 (18)	0.0499 (8)	
N6	0.10563 (13)	−0.2736 (4)	0.88654 (14)	0.0355 (6)	
N7	0.4027 (2)	0.4550 (5)	0.9200 (2)	0.0712 (11)	
N8	0.46565 (16)	−0.1617 (6)	0.83561 (19)	0.0568 (8)	
C1	0.10463 (16)	0.0757 (5)	0.8402 (2)	0.0413 (8)	
H1A	0.0807	0.1668	0.8034	0.050*	
H1B	0.0992	0.1202	0.8924	0.050*	
C2	0.07634 (16)	−0.1309 (6)	0.82762 (19)	0.0448 (8)	
H2A	0.0292	−0.1254	0.8292	0.054*	
H2B	0.0837	−0.1775	0.7763	0.054*	
C3	0.22636 (17)	0.0689 (5)	0.88560 (19)	0.0360 (7)	
H3A	0.2237	0.0584	0.9388	0.043*	
C4	0.20115 (17)	0.0957 (5)	0.76274 (18)	0.0392 (8)	
H4A	0.1767	0.1072	0.7139	0.047*	
C5	0.08740 (15)	−0.3056 (5)	0.95728 (17)	0.0330 (7)	
H5A	0.0522	−0.2435	0.9766	0.040*	
C6	0.15788 (19)	−0.3936 (6)	0.8829 (2)	0.0468 (9)	
H6A	0.1808	−0.4031	0.8399	0.056*	

### Atomic displacement parameters (Å^2^)

**Table:**

	*U*^11^	*U*^22^	*U*^33^	*U*^12^	*U*^13^	*U*^23^
Zn1	0.0323 (3)	0.0385 (3)	0.0298 (2)	−0.00378 (14)	0.00707 (16)	0.00015 (14)
O1	0.080 (2)	0.0613 (18)	0.098 (2)	−0.0275 (16)	0.0486 (19)	−0.0208 (18)
O2	0.142 (4)	0.071 (2)	0.0574 (19)	−0.033 (2)	0.041 (2)	−0.0132 (16)
O3	0.0422 (16)	0.0766 (18)	0.0542 (16)	0.0062 (13)	0.0013 (12)	−0.0270 (14)
O4	0.0506 (18)	0.098 (2)	0.0485 (16)	−0.0159 (16)	0.0018 (13)	−0.0053 (15)
N1	0.0298 (15)	0.0439 (16)	0.0322 (14)	−0.0011 (11)	0.0038 (11)	0.0003 (11)
N2	0.0341 (16)	0.0556 (17)	0.0321 (14)	0.0014 (13)	0.0100 (12)	0.0078 (12)
N3	0.0279 (14)	0.0353 (14)	0.0354 (14)	0.0019 (10)	0.0083 (11)	0.0064 (11)
N4	0.0377 (15)	0.0387 (15)	0.0309 (14)	−0.0026 (11)	0.0097 (12)	−0.0004 (11)
N5	0.052 (2)	0.0539 (17)	0.0480 (18)	0.0126 (15)	0.0243 (15)	0.0090 (15)
N6	0.0336 (14)	0.0440 (15)	0.0293 (13)	−0.0075 (12)	0.0056 (11)	0.0035 (11)
N7	0.110 (3)	0.0459 (19)	0.062 (2)	−0.022 (2)	0.028 (2)	−0.0096 (17)
N8	0.0402 (18)	0.078 (2)	0.0531 (19)	0.0002 (17)	0.0121 (15)	−0.0140 (17)
C1	0.0286 (17)	0.056 (2)	0.0415 (18)	0.0098 (14)	0.0106 (14)	0.0181 (15)
C2	0.0274 (17)	0.074 (2)	0.0320 (17)	−0.0075 (17)	−0.0009 (13)	0.0115 (17)
C3	0.0348 (18)	0.0455 (19)	0.0287 (15)	0.0005 (14)	0.0082 (13)	−0.0004 (13)
C4	0.0374 (19)	0.0522 (19)	0.0285 (16)	0.0049 (15)	0.0060 (14)	0.0088 (14)
C5	0.0317 (17)	0.0392 (16)	0.0290 (15)	−0.0046 (14)	0.0072 (12)	−0.0003 (13)
C6	0.053 (2)	0.053 (2)	0.0378 (18)	0.0008 (17)	0.0219 (16)	0.0017 (16)

### Geometric parameters (Å, °)

**Table:**

Zn1—N4^i^	2.002 (3)	N4—C5	1.307 (4)
Zn1—O1	2.031 (3)	N4—N5	1.386 (4)
Zn1—N1	2.036 (3)	N4—Zn1^i^	2.002 (3)
Zn1—O3	2.046 (3)	N5—C6	1.304 (5)
Zn1—O2	2.477 (3)	N6—C5	1.340 (4)
Zn1—O4	2.488 (3)	N6—C6	1.347 (5)
O1—N7	1.251 (4)	N6—C2	1.471 (4)
O2—N7	1.218 (5)	C1—C2	1.508 (5)
O3—N8	1.276 (4)	C1—H1A	0.9700
O4—N8	1.217 (4)	C1—H1B	0.9700
N1—C3	1.301 (4)	C2—H2A	0.9700
N1—N2	1.388 (4)	C2—H2B	0.9700
N2—C4	1.286 (4)	C3—H3A	0.9300
N3—C3	1.342 (4)	C4—H4A	0.9300
N3—C4	1.350 (4)	C5—H5A	0.9300
N3—C1	1.464 (4)	C6—H6A	0.9300
			
N4^i^—Zn1—O1	128.32 (12)	C5—N6—C6	105.1 (3)
N4^i^—Zn1—N1	103.43 (11)	C5—N6—C2	126.9 (3)
O1—Zn1—N1	107.96 (13)	C6—N6—C2	128.0 (3)
N4^i^—Zn1—O3	119.46 (12)	O2—N7—O1	112.2 (3)
O1—Zn1—O3	97.34 (12)	O4—N8—O3	112.9 (3)
N1—Zn1—O3	95.48 (11)	N3—C1—C2	111.6 (3)
N4^i^—Zn1—O2	88.11 (11)	N3—C1—H1A	109.3
O1—Zn1—O2	52.96 (11)	C2—C1—H1A	109.3
N1—Zn1—O2	89.42 (13)	N3—C1—H1B	109.3
O3—Zn1—O2	149.67 (12)	C2—C1—H1B	109.3
N4^i^—Zn1—O4	86.27 (10)	H1A—C1—H1B	108.0
O1—Zn1—O4	88.80 (12)	N6—C2—C1	112.5 (3)
N1—Zn1—O4	147.07 (10)	N6—C2—H2A	109.1
O3—Zn1—O4	53.47 (10)	C1—C2—H2A	109.1
O2—Zn1—O4	122.68 (12)	N6—C2—H2B	109.1
N7—O1—Zn1	108.1 (2)	C1—C2—H2B	109.1
N7—O2—Zn1	86.7 (2)	H2A—C2—H2B	107.8
N8—O3—Zn1	106.9 (2)	N1—C3—N3	110.1 (3)
N8—O4—Zn1	86.7 (2)	N1—C3—H3A	125.0
C3—N1—N2	107.8 (3)	N3—C3—H3A	125.0
C3—N1—Zn1	132.3 (2)	N2—C4—N3	112.0 (3)
N2—N1—Zn1	120.0 (2)	N2—C4—H4A	124.0
C4—N2—N1	105.6 (3)	N3—C4—H4A	124.0
C3—N3—C4	104.6 (3)	N4—C5—N6	110.0 (3)
C3—N3—C1	127.8 (3)	N4—C5—H5A	125.0
C4—N3—C1	127.6 (3)	N6—C5—H5A	125.0
C5—N4—N5	107.9 (3)	N5—C6—N6	111.6 (3)
C5—N4—Zn1^i^	130.4 (2)	N5—C6—H6A	124.2
N5—N4—Zn1^i^	121.5 (2)	N6—C6—H6A	124.2
C6—N5—N4	105.3 (3)		
			
N4^i^—Zn1—O1—N7	−48.4 (4)	O4—Zn1—N1—N2	−55.0 (3)
N1—Zn1—O1—N7	76.1 (3)	C3—N1—N2—C4	−0.1 (4)
O3—Zn1—O1—N7	174.4 (3)	Zn1—N1—N2—C4	−180.0 (2)
O2—Zn1—O1—N7	1.1 (3)	C5—N4—N5—C6	0.0 (4)
O4—Zn1—O1—N7	−132.7 (3)	Zn1^i^—N4—N5—C6	176.0 (2)
N4^i^—Zn1—O2—N7	142.3 (3)	Zn1—O2—N7—O1	1.5 (4)
O1—Zn1—O2—N7	−1.1 (3)	Zn1—O1—N7—O2	−1.9 (5)
N1—Zn1—O2—N7	−114.3 (3)	Zn1—O4—N8—O3	−0.1 (3)
O3—Zn1—O2—N7	−14.4 (5)	Zn1—O3—N8—O4	0.1 (4)
O4—Zn1—O2—N7	57.9 (3)	C3—N3—C1—C2	−96.2 (4)
N4^i^—Zn1—O3—N8	−59.3 (3)	C4—N3—C1—C2	82.9 (4)
O1—Zn1—O3—N8	82.9 (3)	C5—N6—C2—C1	84.7 (4)
N1—Zn1—O3—N8	−168.1 (3)	C6—N6—C2—C1	−92.2 (4)
O2—Zn1—O3—N8	93.6 (3)	N3—C1—C2—N6	64.9 (4)
O4—Zn1—O3—N8	−0.1 (2)	N2—N1—C3—N3	0.4 (3)
N4^i^—Zn1—O4—N8	131.5 (2)	Zn1—N1—C3—N3	−179.7 (2)
O1—Zn1—O4—N8	−100.0 (3)	C4—N3—C3—N1	−0.6 (3)
N1—Zn1—O4—N8	22.4 (3)	C1—N3—C3—N1	178.7 (3)
O3—Zn1—O4—N8	0.1 (2)	N1—N2—C4—N3	−0.2 (4)
O2—Zn1—O4—N8	−143.1 (2)	C3—N3—C4—N2	0.5 (4)
N4^i^—Zn1—N1—C3	21.0 (3)	C1—N3—C4—N2	−178.7 (3)
O1—Zn1—N1—C3	−117.4 (3)	N5—N4—C5—N6	−0.1 (4)
O3—Zn1—N1—C3	143.0 (3)	Zn1^i^—N4—C5—N6	−175.7 (2)
O2—Zn1—N1—C3	−67.0 (3)	C6—N6—C5—N4	0.3 (4)
O4—Zn1—N1—C3	125.2 (3)	C2—N6—C5—N4	−177.2 (3)
N4^i^—Zn1—N1—N2	−159.2 (2)	N4—N5—C6—N6	0.2 (4)
O1—Zn1—N1—N2	62.4 (2)	C5—N6—C6—N5	−0.3 (4)
O3—Zn1—N1—N2	−37.2 (2)	C2—N6—C6—N5	177.1 (3)
O2—Zn1—N1—N2	112.8 (2)		

Symmetry codes: (i) −*x*+1/2, −*y*−1/2, −*z*+2.

### Hydrogen-bond geometry (Å, °)

**Table:**

*D*—H···*A*	*D*—H	H···*A*	*D*···*A*	*D*—H···*A*
C1—H1B···O2^ii^	0.97	2.53	3.396 (5)	149
C2—H2A···O1^iii^	0.97	2.49	3.417 (4)	160
C3—H3A···O2^ii^	0.93	2.66	3.412 (5)	139
C6—H6A···N2^iv^	0.93	2.39	3.314 (4)	176

Symmetry codes: (ii) −*x*+1/2, −*y*+1/2, −*z*+2; (iii) *x*−1/2, *y*−1/2, *z*; (iv) −*x*+1/2, *y*−1/2, −*z*+3/2.
